# A broadband achromatic metalens array for integral imaging in the visible

**DOI:** 10.1038/s41377-019-0178-2

**Published:** 2019-07-24

**Authors:** Zhi-Bin Fan, Hao-Yang Qiu, Han-Le Zhang, Xiao-Ning Pang, Li-Dan Zhou, Lin Liu, Hui Ren, Qiong-Hua Wang, Jian-Wen Dong

**Affiliations:** 1 State Key Laboratory of Optoelectronic Materials and Technologies, Sun Yat-sen University, Guangzhou, 510275 China; 2 School of Physics, Sun Yat-sen University, Guangzhou, 510275 China; 3 School of Instrumentation and Optoelectronic Engineering, Beihang University, Beijing, 100191 China

**Keywords:** Imaging and sensing, Sub-wavelength optics, Metamaterials

## Abstract

Integral imaging is a promising three-dimensional (3D) imaging technique that captures and reconstructs light field information. Microlens arrays are usually used for the reconstruction process to display 3D scenes to the viewer. However, the inherent chromatic aberration of the microlens array reduces the viewing quality, and thus, broadband achromatic imaging remains a challenge for integral imaging. Here, we realize a silicon nitride metalens array in the visible region that can be used to reconstruct 3D optical scenes in the achromatic integral imaging for white light. The metalens array contains 60 × 60 polarization-insensitive metalenses with nearly diffraction-limited focusing. The nanoposts in each high-efficiency (measured as 47% on average) metalens are delicately designed with zero effective material dispersion and an effective achromatic refractive index distribution from 430 to 780 nm. In addition, such an achromatic metalens array is composed of only a single silicon nitride layer with an ultrathin thickness of 400 nm, making the array suitable for on-chip hybrid-CMOS integration and the parallel manipulation of optoelectronic information. We expect these findings to provide possibilities for full-color and aberration-free integral imaging, and we envision that the proposed approach may be potentially applicable in the fields of high-power microlithography, high-precision wavefront sensors, virtual/augmented reality and 3D imaging.

## Imaging: Metalenses hold promise for new sensing and imaging technologies

A metalens array capable of reconstructing three-dimensional (3D) images has been developed by scientists, opening the door for new applications in microlithography, sensing, and 3D imaging. Integral imaging, where light from an image is reconstructed using a two-dimensional array of microlenses, holds the promise for 3D displays. However, chromatic aberration in the microlens array leads to blurring and color distortion, diminishing image quality. Now, Jian-Wen and colleagues from Sun Yat-sen University in China, working with researches from Beihang University, have developed a metalens array that can reproduce a 3D optical image with achromatic integral imaging for white light. Using a top-down nanofabrication process, the researchers etched a 60 × 60 rectangular metalens array on a layer of silicon nitride deposited onto a quartz substrate, paving the way for their use in high-precision sensors and 3D imaging technologies.

## Introduction

Integral imaging, first proposed in 1908 by Lippmann, is considered to be one of the most promising imaging techniques for 3D displays^1^. The concept was subsequently verified in 1911 by using a pinhole array^2^ and a suitable microlens array in 1948. Replacing the photographic film with a digital device such as a CCD or a LCD^3^ has considerably increased the attraction of integral imaging. Very recently, the possibility of merging integral imaging into a smartphone has been reported by utilizing a matching microlens array^4^. However, even though the microlens array has become a routine component of integral imaging, its inherent chromatic aberration still reduces the viewing quality and prevents full-color imaging from being realized.

Chromatic aberration represents the failure of a lens to focus light of different wavelengths onto the same plane, resulting in blurring and color distortion. Correcting the chromatic aberration in the broadband region is fundamental for achieving full-color imaging. In traditional optical design, several lenses with different focal lengths are usually cascaded together to construct a multiwavelength achromatic lens system. The most common types are achromatic doublet lenses and apochromatic lenses, which are used to focus light of two and three wavelengths, respectively, onto the same focal plane. However, the bulky size of these achromatic lenses limits their applications in portable, wearable and integrated devices. Clearly, replacing these geometrical lenses with more compact components to perform the same achromatic task is imperative. Accordingly, the metalens^5–38^ has recently attracted considerable attention. Numerous studies have already been performed to create achromatic metalenses^24–30^ at several discrete wavelengths. By tailoring the electromagnetic response of nanoposts, a narrow-band achromatic metalens^31^ in the visible region has also been developed. Recently, broadband achromatic metalenses^32–35^ designed by either the group delay method or the plasmonic resonance method have been achieved in the visible region using Pancharatnam–Berry (PB) phase-based nanoposts. Unpolarized broadband achromatic metalenses have also been realized in the near-infrared^36^ and visible^37^ regions with the use of silicon and TiO_2_, respectively. However, a method to fabricate a polarization-insensitive broadband achromatic metalens in a CMOS-compatible way for the visible region has yet to be developed, and hence, relevant research remains highly attractive for certain commercial applications.

Furthermore, since a metalens is composed of only a single layer of subwavelength nanoposts, it can be designed with an ultrasmall aperture to enhance the quality of integral imaging. Very recently, metalenses have been used for light-field imaging^38^, in which an achromatic metalens array is used to capture optical information experimentally and render the imaging on computers with different depths of focus; this constitutes a prospective application of a full-color light-field camera. However, insufficient research has been conducted on the use of metalenses in applications of integral imaging displays. As one of the most important types of 3D displays^39,40^, integral imaging displays encode 3D objects with computational algorithms but reconstruct optical images experimentally in free space; thus, the workflow is somewhat opposite to that of a light-field camera. Since the polarization of light in different display devices are different, metalenses must also be insensitive to the polarization in integral imaging.

**Fig. 1:**
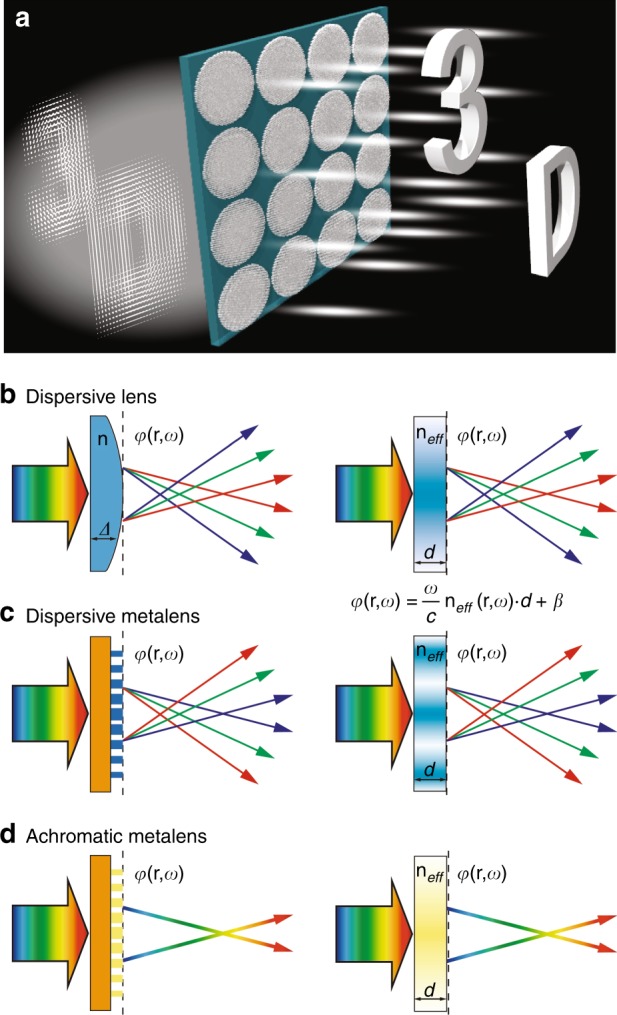
Schematic of the broadband achromatic integral imaging principle and the dispersion properties of a dispersive geometrical lens, a dispersive metalens and an achromatic metalens. **a** Schematic of the broadband achromatic metalens array showing the principle of white-light achromatic integral imaging. **b** Dispersive geometrical lens (left) with normal dispersion in which red light is focused at longer distances than blue light. Its effective slab (right) is shown with an effective refractive index distribution n_*eff*_(r, ω) and a given thickness *d*. The relationship between the exit phase *φ*(r, ω) and n_*eff*_(r, ω) is *φ* = ω/c·n_*eff*_·d + β, where c is the velocity of light and β is a constant. **c** Dispersive metalens (left) and its effective slab (right), which has a longer focal length for blue light than for red light due to the abnormal dispersion. **d** Broadband achromatic metalens (left) and its effective slab (right). Using the effective refractive index method, the n_*eff*_(r, ω) distribution can be deduced by using the known *φ*(r, ω) of a broadband achromatic metalens

Here, we demonstrate a metalens array that can be used to reconstruct the light field experimentally, and we verify the concept of full-color integral imaging (Fig. 1a). We first present a single polarization-insensitive broadband achromatic metalens working in the entire visible region. A fully CMOS-compatible semiconductor material, silicon nitride, is used to design the high-transmission nanoposts with zero effective material dispersion and various values of the effective refractive index, thereby composing the single achromatic metalens. Composed of 60 × 60 metalenses, a broadband achromatic metalens array is then fabricated and used to demonstrate its white-light integral imaging results experimentally.

## Results

### Broadband achromatic method

As is well known, the dispersion of a certain device refers to the derivative of its given performance parameter with respect to the frequency or wavelength; in this article, we use the frequency as the independent variable for convenience. Hence, the dispersion of a lens can be defined as the derivative of the focal length versus the frequency d*f*/d*ω*. A conventional geometrical lens has a normal dispersion (d*f*/d*ω* < 0, Fig. 1b), indicating that blue light is deflected more than red light, while a diffractive lens or common metalens follows the anomalous dispersion (d*f*/d*ω* > 0, Fig. 1c) insomuch that the focal length for blue light is larger than that for red light. A perfect achromatic lens, required to generate a full-color image, must be dispersionless (d*f*/d*ω* = 0, Fig. 1d) and must be able to focus light at any wavelength within the working range onto the same focal plane. In our opinion, all these lens components can be effectively viewed as a relevant slab (Fig. 1b–d right) of uniform thickness under certain conditions. In the following, we propose a broadband achromatic method from a novel perspective of the effective refractive index, providing a more accessible manner with which to understand the impacts of dielectric materials on broadband achromatic design.

**Fig. 2:**
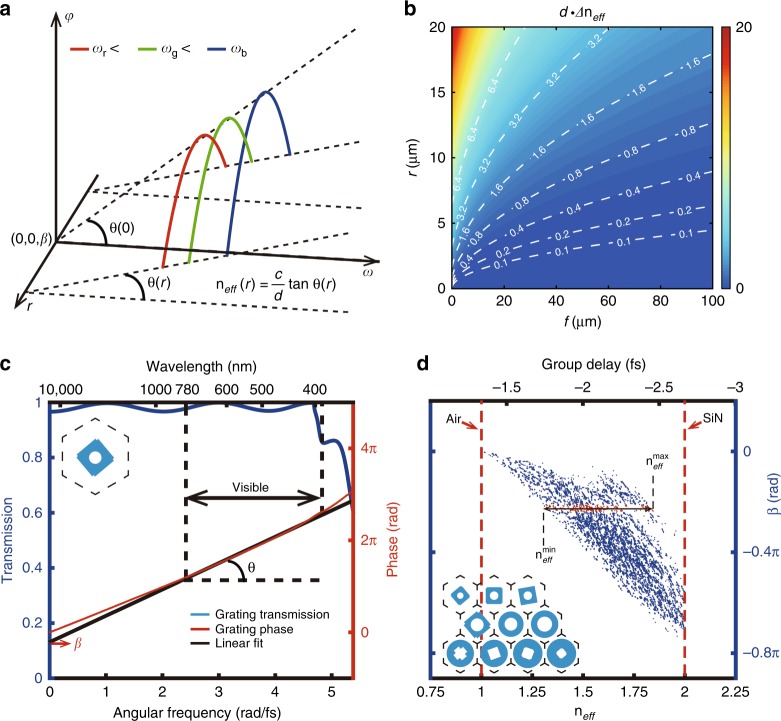
Design of a broadband achromatic metalens. **a** Sketch map of the phases of a broadband achromatic lens in the r-ω space with a fixed focal length *f*. The coordinates of the original point (r, ω, *φ*) are set as (0, 0, β). Three-phase profiles at 430, 530, and 630 nm are plotted. The value of α is set as 1.45 μm, and β is −1.25 rad. For a given radius of the metalens r, the achromatic phase *φ* should be linearly associated with the frequency ω, indicating that the effective refractive index n_*eff*_ is independent of the frequency via the relationship between the slant angle θ(r) and n_*eff*_. **b** Effective optical path difference (OPD) *d∆*n_*eff*_ in the *f*−r space. Several values of the effective OPD are plotted as white curves (Unit, μm). Note that a broadband achromatic metalens with a large radius and short focal length needs a large effective OPD *d∆*n_*eff*_, e.g., the red color in the upper-left corner. **c** Transmission (blue) and phase (red) of a silicon nitride grating. The inset shows the geometry of the nanoposts, where the diameter of the circular hole is 60 nm. The hexagonal lattice constant and the thickness are 320 nm and 400 nm, respectively. A high transmission and a near-linear phase as a function of the angular frequency are achieved in the entire visible region. A pair of parameters (n_*eff*_, β) is then retrieved from the linear fitting of the phase (black), where n_*eff*_ = c/*d*·tan(θ) and β is the crossing point at zero frequency. **d** Calculated (n_*eff*_, β) results for all considered silicon nitride gratings. Each blue point represents a different grating. Red dashed lines indicate the air and silicon nitride materials; the results show that the n_*eff*_ values of all considered gratings are mostly located between the background material (air) and the chosen dielectric material (SiN). By selecting the optimal β value to obtain the maximum *∆*n_*eff*_, the gratings at red points can be screened out; finally, ten kinds of gratings (inset) are chosen after the discretization of n_*eff*_ to compose the broadband achromatic metalens

Previous studies used the phase profile of $\varphi \left( {{\mathrm{r,}}\omega _0} \right) = \omega _0\Gamma \left( {{\mathrm{r,}}\omega _0} \right)$, where *Γ* is a function of the desired performance, to create a metasurface device at a given frequency. To consider the broadband design of a metasurface device, we need to generalize the phase profile into a more general form for a continuous frequency region while maintaining the desired performance at each frequency. Since the phase distribution is relative to a reference point at a given position r and frequency *ω*, we can rewrite the phase profiles in the following form by adding two constants, namely, *α* and *β*, thereby guaranteeing that the performance of the metasurface device will not change, yielding $\varphi \left( {{\mathrm{r,}}\omega } \right) = \omega \left[ {\Gamma \left( {{\mathrm{r,}}\omega } \right) + \alpha } \right] + \beta$. Hence, the relative phase is provided by the metalens elements with respect to the following:

1 $$\varphi \left( {{\mathrm{r,}}\omega } \right) = \frac{\omega }{{\it{c}}}\left( { - \sqrt {{\mathrm{r}}^2 + {\it{f}}^2} + {\it{f}} + \alpha } \right) + \beta$$

where *f* is the focal length and *c* is the velocity of light in vacuum; for convenience, *c* is placed outside the bracket. Equation (1) implies that *β* represents the spectral reference phase at the zero frequency (wavelength λ → ∞), and *α* decides the spatial reference phase at the center of the lens (*r* = 0). Therefore, broadband achromatic elements can be achieved when the phase profiles satisfy the form of Equation (1) with a fixed focal length at any working frequency. Figure 2a plots a sketch map of three-phase profiles (blue, green, and red light) in the r-ω space. When neglecting reflection and considering only the phase of transmitted light, the metalens can be regarded as a slab (Fig. 1) with a fixed thickness *d* and an inhomogeneous effective refractive index n_*eff*_; therefore, Equation (1) can be rewritten as

$$\varphi \left( {{\mathrm{r,}}\omega } \right) = \frac{\omega }{{\it{c}}}n_{{\it{eff}}}\left( {{\mathrm{r,}}\omega } \right) \cdot {\it{d}} + \beta ,$$

2 $${\mathrm{where}}\,n_{{\it{eff}}}\left( {{\mathrm{r,}}\omega } \right) = \frac{1}{{\it{d}}}\left( { - \sqrt {{\mathrm{r}}^2 + {\it{f}}^2} + {\it{f}} + \alpha } \right)$$

For a dispersionless metalens (d*f* /d*ω* = 0), we have

3 $$\frac{\partial }{{\partial \omega }}n_{{\it{eff}}}\left( {{\mathrm{r,}}\omega } \right) = 0\;,\quad \quad \omega \in \left[ {\omega _{{\mathrm{min}}},\;\omega _{{\mathrm{max}}}} \right]$$

The left-hand side of Equation (3) represents the effective material dispersion. That is, the zero effective material dispersion is necessary for the broadband achromatic metalens, and the achromatic bandwidth *Δω* *=* *ω*_max_ - *ω*_min_ depends on the spectrum range sustained by the zero effective material dispersion. As n_*eff*_ is independent of the frequency, the phase must change linearly with the frequency according to Equation (2). Through a simple derivation, we can further acquire the relationship $n_{{\it{eff}}} = \frac{{\it{c}}}{{\it{d}}} \cdot \frac{{\partial \varphi }}{{\partial \omega }} = - \frac{{\it{c}}}{{\it{d}}}\tau _{\mathrm{g}} = \frac{{\it{c}}}{{\it{d}}}{\mathrm{tan}}\;\theta$, where τ_g_ is the group delay and θ is the slant angle (Fig. 2a) of the intersection between the phase and *ω*-axis. Note that the above formula not only gives the relationship between n_*eff*_ and the group delay τ_g_ but also provides a way to calculate the value of n_*eff*_ through the slant angle θ.

The aforementioned text discusses the bandwidth restriction on the achromatic metalens. The size limitation can be analyzed from Equation (2). At the center (*r* = 0) of the metalens, we have $n_{{\it{eff}}}^{{\mathrm{max}}} = \frac{\alpha }{{\it{d}}}$. For the whole metalens, we have ${\it{\Delta }}n_{{\it{eff}}} = \frac{1}{{\it{d}}}\left( {\sqrt {{\mathrm{r}}_{{\mathrm{max}}}^2 + {\it{f}}^2} - {\it{f}}} \right)$. Note that the maximum n_*eff*_ is reached at the center of the metalens, and the constant *α* represents the maximum effective optical path (OP) *d*·n_*eff*_. The maximum size r_max_ of the metalens depends on the effective optical path difference (OPD) *d*·*∆*n_*eff*_. Figure 2b shows a map of the *d*·*∆*n_*eff*_ that the achromatic metalens needs. For a given focal length, a larger aperture achromatic metalens requires a larger effective OPD value by either increasing the metalens thickness *d* or enlarging the range of the n_*eff*_ distribution. However, the size of an achromatic metalens is always restricted on a small scale due to the difficulty of fabricating high-ratio nanoposts and the limitation on the n_*eff*_ range. For example, for silicon nitride (*n* = 2) and air (*n* = 1) materials and for a given thickness *d* = 0.8 μm, the maximum *d*·*Δ*n_*eff*_ is 0.8 μm. Figure 2b shows that, in principle, the radius of an achromatic metalens with a focal length of 50 μm can only be less than 9 μm. As a whole, to create a broadband achromatic metalens, one needs to achieve zero effective material dispersion in the spectral region and to enlarge the range of the n_*eff*_ distribution as much as possible in the spatial region.

### Design of the broadband achromatic metalens

As mentioned above, we need to design nanoposts to achieve zero effective material dispersion. For our purpose of manipulating unpolarized visible light, nanoposts with 90° spatial rotational symmetries are given priority over other nanoposts. Here, a hexagonal silicon nitride grating with a subwavelength lattice constant (*a* = 320 nm) on a silica substrate is considered. The basic geometrical shape is shown in the inset of Fig. 2c, where the diameter of the circular hole is 60 nm. The thickness of the silicon nitride grating is set as 400 nm. Figure 2c plots the simulation results of the transmission and phase spectra for normal incidence light with y-polarization calculated by using rigorous coupled-wave analysis (RCWA)^41^. The blue curve shows that high transmission can be kept well over an ultralarge spectral region, in which the wavelength λ is greater or far greater than the lattice constant *a*. When the wavelength is near or less than the lattice constant, the transmission drops sharply. The red curve shows that the phase increases almost linearly in the visible region, implying that zero effective material dispersion can be achieved in such frequency regions. After a linear fitting calculation (black line), one can obtain the frequency-independent n_*eff*_ from the slant angle θ and the parameter *β* of such a nanopost. In this way, a pair of values (n_*eff*_, *β*) can be used to describe the nearly linear phase spectrum over the entire visible region for a single kind of nanopost. One simple way to enlarge the range of the n_*eff*_ distribution is to calculate these values for as many as types of nanoposts with different structural parameters as possible (Fig. S2). As illustrated in Fig. 2d, the red dashed lines correspond to the air and silicon nitride materials. Each point in Fig. 2d represents a different nanopost, and all points are located between the air and silicon nitride lines, implying that the n_*eff*_ values of all considered nanoposts cannot fall outside the scope between the chosen dielectric material and the background material. Then, the value of the constant *β* is chosen to achieve the maximum *Δ*n_*eff*_, highlighted by the red points in Fig. 2d. Finally, ten kinds of nanoposts (inset of Fig. 2d) are selected to map different values of n_*eff*_ and are placed on the silicon dioxide substrate to form a hexagonal lattice with a subwavelength lattice constant *a*. It is worth noting that there is no longer a need to set a given frequency in the design process because the n_*eff*_ values of all selected nanoposts are independent of the frequency throughout the working region. This constitutes a considerable difference between the previously proposed metalenses and the proposed achromatic metalens.

### Characteristics of the broadband achromatic metalens

**Fig. 3:**
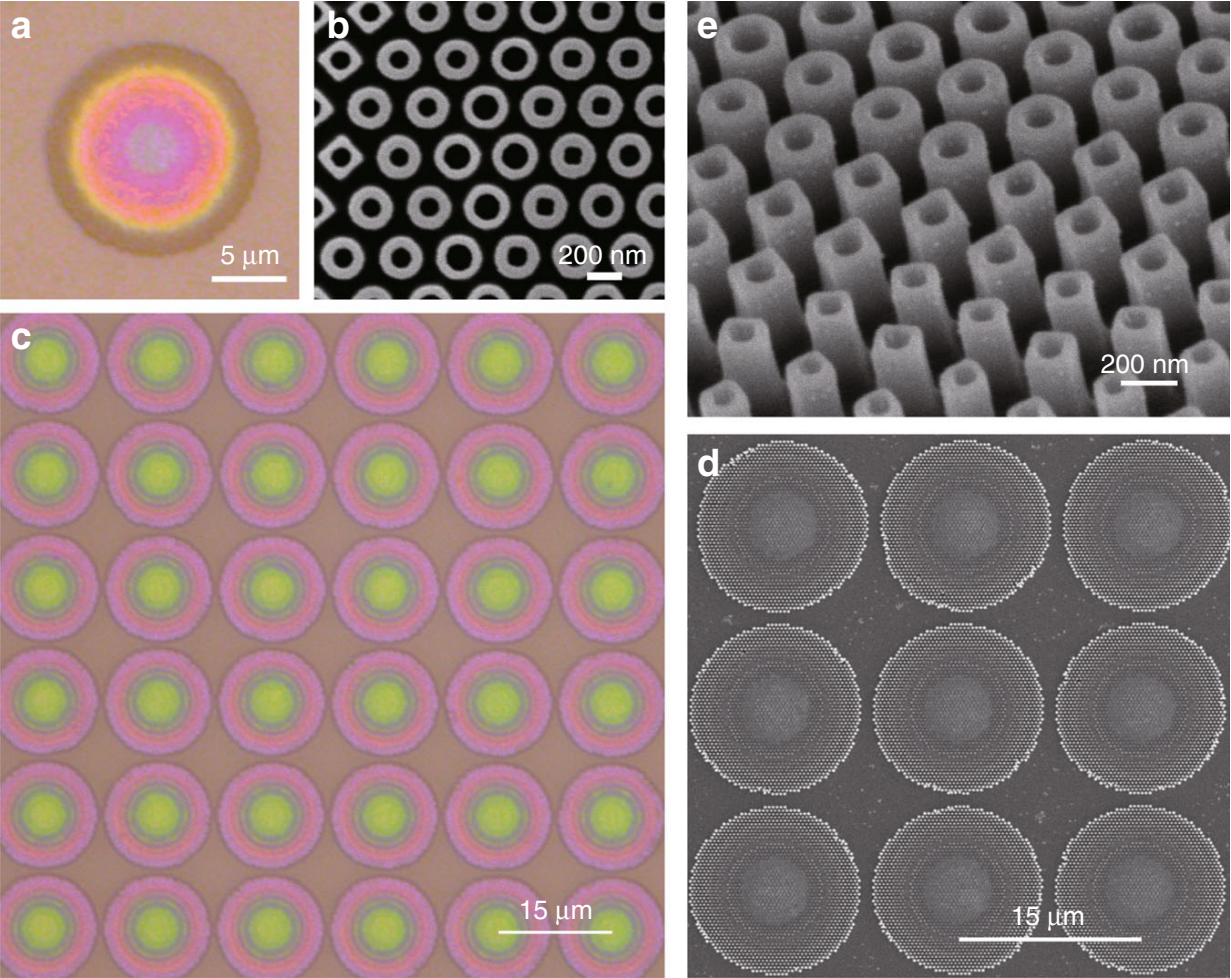
Optical and scanning electron microscope (SEM) images of a single broadband achromatic metalens and metalens array. **a** Optical image of a single metalens. Scale bar: 5 μm. **b** Top-view SEM image of a portion of the fabricated single metalens shown in (**a**). Scale bar: 200 nm. **c** Optical image of a broadband achromatic metalens array. Scale bar: 15 μm. **d** Top-view SEM image of a portion of the fabricated metalens array shown in **c**. Scale bar: 15 μm. **e** Side-view SEM image of a portion of the fabricated metalens array shown in (**c**). Scale bar: 200 nm

**Fig. 4:**
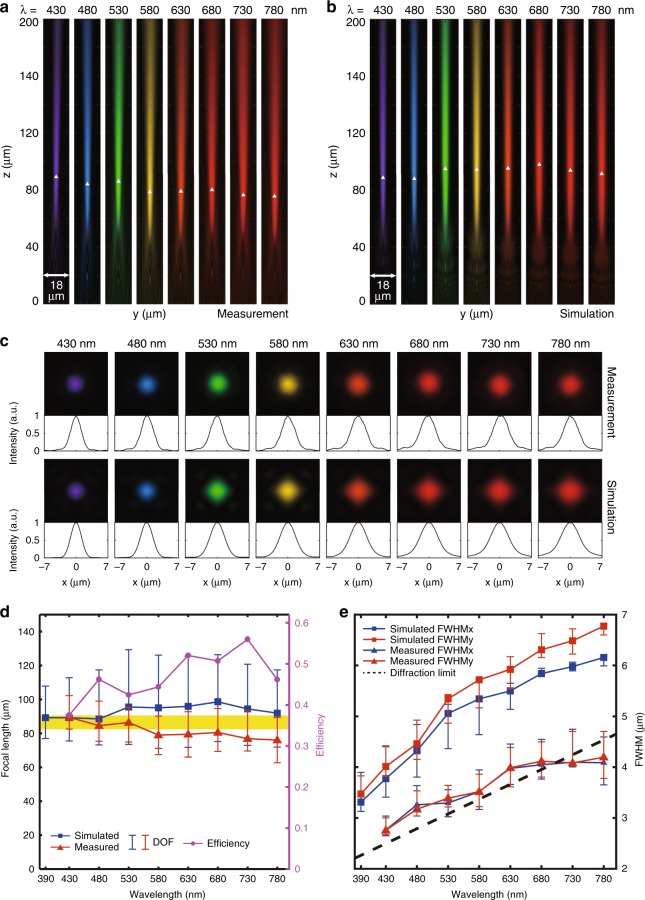
Measurement and simulation results for a single broadband achromatic metalens with a diameter of 14 μm. **a** Measured normalized intensity distributions on a linear scale in the y–z plane using pseudo colors corresponding to their respective wavelengths. The wavelengths of incidence are denoted on the top. The focal points are marked by white triangles. **b** Corresponding simulated normalized intensity distributions. **c** Measured and simulated normalized intensity distributions on a linear scale in the focal plane. **d** Measured (blue square points) and simulated (red triangle points) focal lengths at different wavelengths. The average measured focal length is approximately 81.5 μm, yielding an NA of 0.086. The error bars show the depth of focus (DOF), which is defined here as the range around the focus at which the normalized intensity has fallen to 0.95. Since the metalens can still effectively perform imaging inside the DOF, an achromatic region (yellow area) can be obtained by analyzing the error bars at all wavelengths The purple dotted line shows that the measured focusing efficiency can be maintained in the range of 36% ~ 55% at the measured wavelengths, while the maximum is reached at 730 nm. **e** Measured (triangles) and simulated (squares) full width at half maximums (FWHMs) at different wavelengths. The blue color denotes the results in the x-direction, while the red color denotes the results in the y-direction. The error bars show the ranges of the FWHM inside the DOF regions

Figure 3a, b illustrate optical and scanning electron microscope (SEM) images, respectively, of the proposed achromatic metalens with a diameter of 14 μm. The metalens sample is achieved by a top-down nanofabrication process via standard electron-beam lithography (EBL) technique. The top-view SEM image (Fig. 3b) of a portion of the metalens with a relatively high magnification shows the well-fabricated nanoposts with circular or square holes. The measurement and simulation results of the field distributions between 430 and 780 nm are shown in Fig. 4a, b. The y-polarized plane wave is normally incident onto the metalens from the silicon dioxide substrate as measurement setup (Fig. S3) shown. Visible light can be focused approximately onto the same focal plane with a relatively long focal depth. Figure 4c displays the measured and simulated normalized intensity distributions on a linear scale in the focal plane. The related x-direction cross-sections are shown on the bottom. The simulated (blue squares) and measured (red triangles) focal lengths are plotted in Fig. 4d. One can see that the average measured focal length is ~ 81.5 μm, yielding an experimental NA of 0.086. To describe the focal depth, we define the depth of focus (DOF) here as the range around the focus at which the normalized intensity has fallen to 0.95 and use an error bar to denote it. Analyzing the DOF at all measured wavelengths, an overlapping region from *z* = 82.5 to 89.4 μm can be obtained (yellow in Fig. 4d). Such a region is regarded as the achromatic focusing region of the proposed single metalens. The purple dotted line shows that the measured focusing efficiency can be maintained in the range of 36% ~ 55% at all visible wavelengths. An average efficiency of 47% is achieved, and the maximum is reached at 730 nm. The full width at half maximums (FWHMs) at different wavelengths are also plotted in Fig. 4e, and the corresponding error bars denote the ranges of the FWHM within the DOF regions. It can be seen that the FWHMs increase with the wavelengths in both the simulation and the measurement results; additionally, with the nearly diffraction-limited spot (λ/2NA), the measured values are lower than the simulated values because the actual measured focal lengths are shorter.

### Achromatic metalens array and integral imaging

**Fig. 5:**
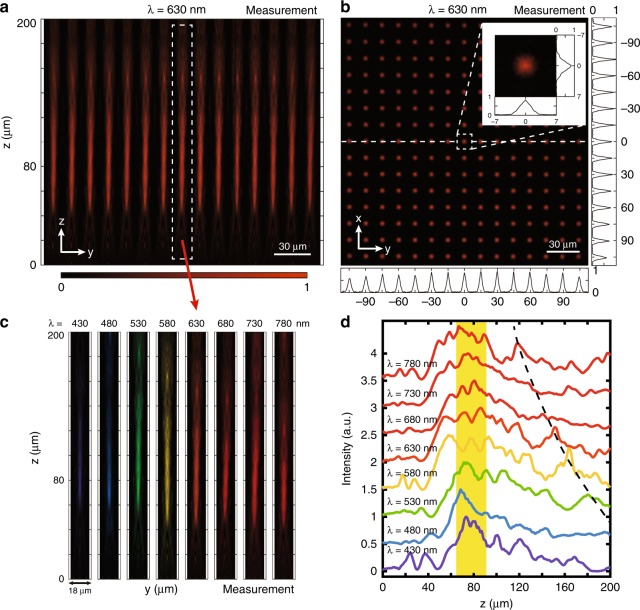
Measurement results for a broadband achromatic metalens array. The diameter of each metalens is 14 μm, and the lattice constant between adjacent metalenses is 15 μm. **a** Normalized intensity distribution on a linear scale in the y–z plane at 630 nm. The field distributions of 15 metalenses are collected, and the most notable feature is that each metalens shows the uniformity of each focusing effect, validating the high-quality fabrication process. Compared with the corresponding result for a single metalens at 630 nm in Fig. 4a, the focal point with a long DOF splits into several main peaks around z = 80 μm, and a secondary feeble focal point appears at z ≈ 150 μm. **b** Normalized intensity distributions on a linear scale in the focal x–y plane at 630 nm. Each metalens focuses well in its own area. The black lines on the bottom and right show the y-direction and x-direction cross sections, respectively, of the array center. The white line in the center indicates the cross section used in (**a**). The inset illustrates the field distribution of the central metalens with a larger magnification. **c** Normalized intensity distributions of the middle metalens (red arrow) within the array on a linear scale in the y–z plane using false colors corresponding to different wavelengths. **d** Z-direction cross sections in (**c**) at all wavelengths. The metalens array mainly focuses amounts of energy on the same yellow region around z = 80 μm, indicating that the broadband achromatic property has been achieved. Weak secondary focal points caused by the periodic arrangement of metalenses also emerge with the abnormal dispersion insomuch that the secondary focal length decreases as the wavelength increases (black dashed line). However, such secondary focal points are too weak to affect the achromatic property

To further demonstrate our purpose, we take the metalens as a subsystem by constructing a metalens array. We first fabricate an achromatic metalens array composed of 15 × 15 metalenses as discussed above; the optical and SEM images are shown in Fig. 3c–e. The side-view SEM image of the metalens in Fig. 3e describes the vertical profile of the nanoposts. Figure 5a, b show the measured normalized intensity distributions on a linear scale in the y–z plane and the focal x–y plane at 630 nm, respectively. It is obvious in Fig. 5a that each metalens demonstrates the uniformity of each focusing effect. Compared with the results for a single metalens in Fig. 4a, the focal point with a long DOF splits into several main peaks around *z* = 80 μm, and a secondary focal point appears at *z* = 150 μm. A 15 × 15 spot array is shown in Fig. 5b, implying that each metalens focuses well on its own area. The cross-sections in the y- and x-directions are plotted on the bottom and right of Fig. 5b, respectively, while the intensity distribution of the central metalens is illustrated with a larger magnification (inset). Furthermore, the achromatic focusing effect of the central metalens (red arrow) is also illustrated in Fig. 5c, and the z-direction cross sections at different wavelengths are plotted in Fig. 5d. Note that the metalens array mainly focuses on the whole visible spectrum within the same region around z = 80 μm (yellow area), implying that the broadband achromatic property is achieved. A weak secondary focal point, caused by the periodic arrangement of metalenses, also emerges with the abnormal dispersion insomuch that the secondary focal length decreases as the wavelength increases (black dashed line). However, due to the long secondary focal length and the feeble intensity (values less than 0.5 at most measured results), the influence of this secondary focal point is weak for the achromatic property.

**Fig. 6:**
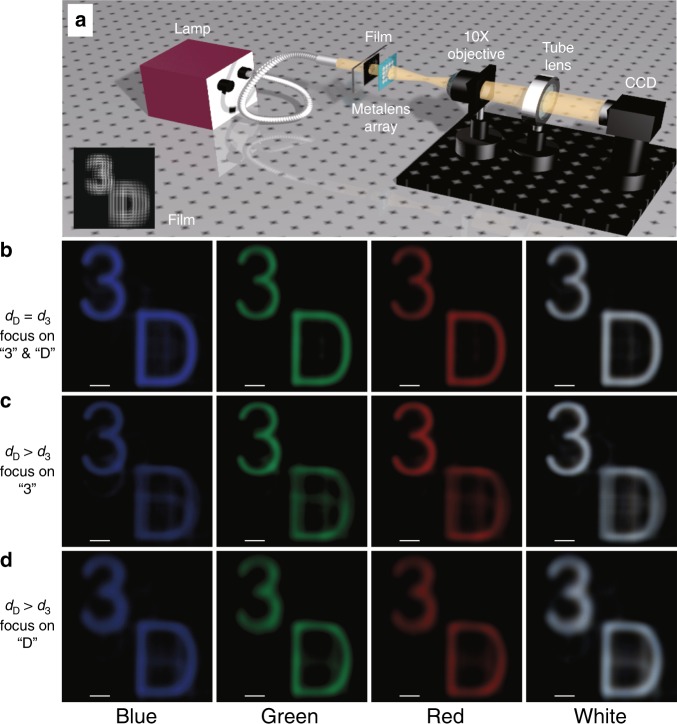
Imaging demonstration of the broadband achromatic metalens array. **a** Measurement configuration for the imaging demonstration of the broadband achromatic metalens array. The object films illuminated by blue, green, red and white light from the lamp and their reconstructed images can be captured by the following microscopy imaging system. Blue, green and red light is generated by inserting narrow-band (10 nm) filters of 488 nm, 532 nm and 633 nm, respectively. **b** Reconstructed images in the case of *d*_D_ = *d*_3_, which means that the number “3” and the letter “D” are on the same depth plane relative to the central depth plane. The characters “3” and “D” will always become clear together in this case. Good-quality images, especially in the white-light case, can be observed by adjusting the film and metalens array to match each other. The white-light image explicitly shows the broadband achromatic property of the metalens array in the entire visible region. Scale bar, 100 μm. **c**, **d** Reconstructed images in the case of *d*_D_ > *d*_3_, which means that the number “3” is closer to the central depth plane than the letter “D”, and they are on different depth planes (Fig. S5b shows the sketch map). Hence, when we move the microscopy imaging system along the optical axes to focus on the number “3” (as shown in **c**), the letter “D” continues to blur since they are not on the same depth plane. When we focus on the letter “D” in (**d)**, the number “3” will become blurred compared with that in (**c**). These imaging results effectively demonstrate the three-dimensional image depth using our metalens array in integral imaging. Scale bar, 100 μm

To better demonstrate the imaging quality, we further fabricate another array of 60 × 60 broadband achromatic metalenses. The measurement configuration for this imaging demonstration is shown in Fig. 6a, and the imaging results are shown in Fig. 6b–d. The object film is an elemental image array calculated by the computational algorithm using the integral imaging acquisition principle^42^ (Supplementary material S4). The light from the 3D object (the number “3” and the letter “D” on different planes) passes through an ideal achromatic microlens array to form the elemental image array. The object film illuminated by the blue, green, red and white light from the lamp and the corresponding reconstructed images can be captured by the following microscopy system. Blue, green and red light is generated by inserting narrow-band (10 nm) filters of 488, 532, and 633 nm, respectively. In these experiments, we adjust the light path system to make the image clear and then change only the narrow-band filter to another filter or remove it entirely to observe the imaging qualities for light of different colors. Here, we consider two cases to show the optical reconstruction effect. In the computer modeling process, we set the distance between the number “3” (letter “D”) and the central depth plane as *d*_3_ (*d*_D_), as shown in Fig. S4. In the first case of *d*_D_ = *d*_3_, the two characters are on the same depth plane (Fig. S5a), and hence, they will always become clear together (Fig. 6b). It is obvious that the characters can be reconstructed well in blue, green and red light and even white light. This is powerful evidence for the realization of our broadband achromatic metalens array over the entire visible region. To further show the image depth effect, we set *d*_D_ > *d*_3_ in the second case, which means that the number “3” is closer to the central depth plane than is the letter “D” (Fig. S5b). When we move the microscopy system along the optical axes to focus on the number “3” (Fig. 6c), the letter “D” continues to blur. On the other hand, when we focus on the letter “D” (Fig. 6d), the number “3” will become blurred compared with that in Fig. 6c. We also provide videos of these two cases in the supplementary material. One can clearly see the unambiguous in-focus/blurry effect when the reconstruction plane locates different image depths. Video m1 for the case of *d*_D_ = *d*_3_ shows that both characters become clear or blurred together, while Video m2 for the case of *d*_D_ > *d*_3_ illustrates that the number “3” becomes clear first, followed by the letter “D”. These imaging results verify the 3D image depth effect using our metalens array in integral imaging.

## Discussion

By designing the geometries of nanoposts, a series of polarization-insensitive silicon nitride gratings are obtained by using zero effective material dispersion and different values of the effective refractive index. According to the achromatic effective refractive index distribution, a transmissive achromatic metalens is realized in the visible spectrum from 430 to 780 nm. By composing the metalenses in a rectangular lattice, a 60 × 60 achromatic metalens array is achieved. We also show the broadband achromatic integral imaging performance with a white-light source. Finally, the low-cost and CMOS-compatible fabrication process makes the achromatic metalens array suitable for numerous applications, such as microlithography, wavefront sensors, and 3D imaging.

## Materials and methods

### Imaging setup

In the experimental setup (Fig. 6a), a white-light source (Thorlabs OSL2) is applied to illuminate the metal film, and a series of narrow-band (10 nm) filters (not shown in the light path) with different center wavelengths are placed between the lamp and the film. A microscopy imaging system composed of a ×20 objective lens (Olympus MPLFLN20xBD), a tube lens (Thorlabs TTL180) and a color CCD camera (Mshot MC20) is used to observe the images. Note that such a microscopy imaging system is mounted on a large translation stage for convenient movement.

## Supplementary information

SUPPLEMENTARY MATERIAL

Video m1

Video m2

## References

[1] Lippmann, G. La photographie intégrale. *Comtes Rendus*, *Academie des Sciences*. 446–451 (1908).

[2] Sokolov, A. P. Autostereoscopy and Integral Photography by Professor Lippmann’s Method. (IZD MGU: Moscow State University Press, 1911).

[3] Okano F (1997). Real-time pickup method for a three-dimensional image based on integral photography. Appl. Opt..

[4] Ren H (2019). Super-multiview integral imaging scheme based on sparse camera array and CNN super-resolution. Appl. Opt..

[5] Aieta F (2012). Aberration-free ultrathin flat lenses and axicons at telecom wavelengths based on plasmonic metasurfaces. Nano Lett..

[6] Xu HX (2016). Aberration-free and functionality-switchable meta-lenses based on tunable metasurfaces. Appl. Phys. Lett..

[7] Chen K (2017). A Reconfigurable active huygens’ metalens. Adv. Mater..

[8] West PR (2014). All-dielectric subwavelength metasurface focusing lens. Opt. Express.

[9] Arbabi A (2015). Subwavelength-thick lenses with high numerical apertures and large efficiency based on high-contrast transmitarrays. Nat. Commun..

[10] Li RZ (2016). Broadband, high-efficiency, arbitrary focusing lens by a holographic dielectric meta-reflectarray. J. Phys. D: Appl. Phys..

[11] Verslegers L (2009). Planar lenses based on nanoscale slit arrays in a metallic film. Nano Lett..

[12] Chen XZ (2015). Longitudinal multifoci metalens for circularly polarized light. Adv. Opt. Mater..

[13] Ni XJ (2013). Ultra-thin, planar, Babinet-inverted plasmonic metalenses. Light.: Sci. Appl..

[14] Chen XZ (2012). Dual-polarity plasmonic metalens for visible light. Nat. Commun..

[15] Chen XZ (2013). Reversible three-dimensional focusing of visible light with ultrathin plasmonic flat lens. Adv. Opt. Mater..

[16] Khorasaninejad M (2016). Metalenses at visible wavelengths: diffraction-limited focusing and subwavelength resolution imaging. Science.

[17] Khorasaninejad M (2016). Polarization-insensitive metalenses at visible wavelengths. Nano Lett..

[18] Chen WT (2017). Immersion meta-lenses at visible wavelengths for nanoscale imaging. Nano Lett..

[19] Groever B, Chen WT, Capasso F (2017). Meta-lens doublet in the visible region. Nano Lett..

[20] Zhan AL (2017). Metasurface freeform nanophotonics. Sci. Rep..

[21] Zhou JX (2018). Broadband photonic spin hall meta-lens. ACS Nano.

[22] Fan ZB (2018). Silicon nitride metalenses for close-to-one numerical aperture and wide-angle visible imaging. Phys. Rev. Appl..

[23] Colburn S, Zhan AL, Majumdar A (2018). Metasurface optics for full-color computational imaging. Sci. Adv..

[24] Khorasaninejad M (2015). Achromatic metasurface lens at telecommunication wavelengths. Nano Lett..

[25] Aieta F (2015). Multiwavelength achromatic metasurfaces by dispersive phase compensation. Science.

[26] Arbabi E (2016). Multiwavelength polarization-insensitive lenses based on dielectric metasurfaces with meta-molecules. Optica.

[27] Arbabi E (2016). High efficiency double-wavelength dielectric metasurface lenses with dichroic birefringent meta-atoms. Opt. Express.

[28] Eisenbach O (2015). Metasurfaces based dual wavelength diffractive lenses. Opt. Express.

[29] Zhao ZY (2015). Multispectral optical metasurfaces enabled by achromatic phase transition. Sci. Rep..

[30] Arbabi E (2016). Multiwavelength metasurfaces through spatial multiplexing. Sci. Rep..

[31] Khorasaninejad M (2017). Achromatic metalens over 60 nm bandwidth in the visible and metalens with reverse chromatic dispersion. Nano Lett..

[32] Arbabi E (2017). Controlling the sign of chromatic dispersion in diffractive optics with dielectric metasurfaces. Optica.

[33] Wang SM (2017). Broadband achromatic optical metasurface devices. Nat. Commun..

[34] Wang SM (2018). A broadband achromatic metalens in the visible. Nat. Nanotechnol..

[35] Chen WT (2018). A broadband achromatic metalens for focusing and imaging in the visible. Nat. Nanotechnol..

[36] Shrestha S (2018). Broadband achromatic dielectric metalenses. Light.: Sci. Appl..

[37] Chen WT (2019). A broadband achromatic polarization-insensitive metalens consisting of anisotropic nanostructures. Nat. Commun..

[38] Lin RJ (2019). Achromatic metalens array for full-colour light-field imaging. Nat. Nanotechnol..

[39] Wang QH (2016). Dual-view integral imaging 3D display by using orthogonal polarizer array and polarization switcher. Opt. Express.

[40] Wang XR, Hua H (2008). Theoretical analysis for integral imaging performance based on microscanning of a microlens array. Opt. Lett..

[41] Liu V, Fan SH (2012). S^4^: a free electromagnetic solver for layered periodic structures. Comput. Phys. Commun..

[42] Li SL (2014). Multiple orthographic frustum combing for real-time computer-generated integral imaging system. J. Disp. Technol..

