# Supplementary material for: A broadband achromatic metalens array for integral imaging in the visible
Source: Light Sci Appl. 2019 Jul 24;8:67. doi: 10.1038/s41377-019-0178-2 (PMC6804934; doi:10.1038/s41377-019-0178-2)
Supplement: Supplementary file 1 — SUPPLEMENTARY MATERIAL [file 41377_2019_178_MOESM1_ESM.pdf]

SUPPLEMENTARY MATERIAL

A broadband achromatic metalens array for integral imaging in the visible

Zhi-Bin Fan[1,2,†], Hao-Yang Qiu[1,2,†], Han-Le Zhang[3,†], Xiao-Ning Pang[1,2], Li-Dan Zhou[1], Lin Liu[1], Hui Ren[3], Qiong-Hua Wang[3,*], Jian-Wen Dong[1,2,*]

[1]*State Key Laboratory of Optoelectronic Materials and Technologies, Sun Yat-sen University, Guangzhou 510275, China.*

[2]*School of Physics, Sun Yat-sen University, Guangzhou 510275, China.*

[3]*School of Instrumentation and Optoelectronic Engineering, Beihang University, Beijing 100191, China.*

†*These authors contributed equally to this work.*

**Correspondence: QH Wang, E-mail: qionghua@buaa.edu.cn;*

JW Dong, E-mail: dongjwen@mail.sysu.edu.cn.

8 pages, 6 figures S1-S6

S1: Fabrication

The metalens array samples were achieved by a top-down nanofabrication process shown in the diagrammatic drawing (Fig. S1). First, a 400-nm-thick silicon nitride film is deposited on the quartz substrate at the temperature of 300°C with the appropriately optimized hydrogen concentration and film stress by the inductively coupled plasma-chemical vapor deposition (ICP-CVD) system (Plasmalab System 100 ICP180, Oxford). After removing the residual organics on the film, the positive electron-beam resist (ARP6200.09) is spin-coating on the silicon nitride film. The metalens patterns are then defined in this high-resolution positive resist by an electron-beam lithography (EBL) system (Vistec EBPG5000 ES) at 100 KV, and subsequently developed by the dimethylbenzene for about 100 seconds. Next, the reactive-ion-etch (RIE) (Oxford Instrument Plasmalab System 100 RIE180) with a mixture of CHF_3 and O_2 gases is applied to etch through the 400-nm-thick silicon nitride layer. Finally, the residual resist is removed by the dry etching with the O_2 plasma descum, and hence the silicon nitride nanoposts are patterned as the desired metalens array.

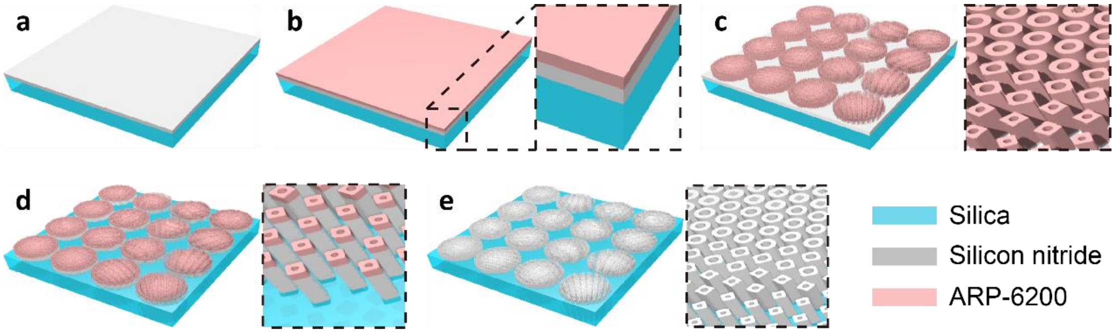

Figure S1. Schematic illustration of fabrication. a Deposition of 400-nm-thick silicon nitride on the quartz substrate; **b** The spin-coating photoresist of ARP6200; **c** Transferring patterns onto ARP6200 by EBL; **d** Etching through the 400-nm-thick silicon nitride layer; **e** Removing the residual resist by dry etching.

S2: Geometries of silicon nitride nanoposts

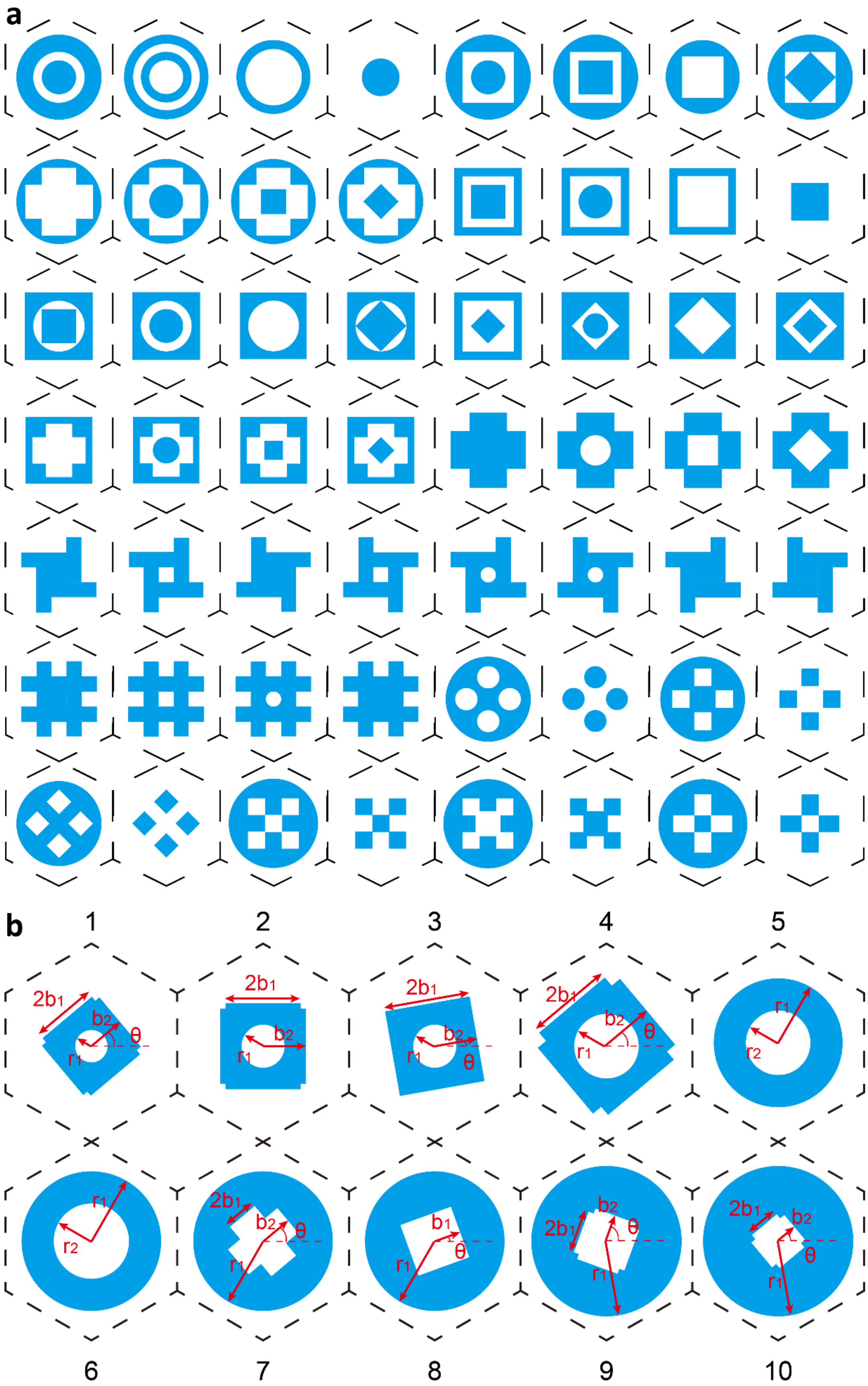

Figure S2. Geometries of silicon nitride nanoposts. a All types of nanoposts in the considered silicon nitride gratings in the main text in Fig. 2d. The spectral responses of silicon nitride gratings for each nanopost type with different geometrical parameters have been calculated to get the value of (n_{eff}, β). **b** The selected nanoposts for the broadband achromatic metalens array. The parameters of all selected nanoposts are listed in Table S1.

Table S1. The parameters of all selected nanoposts.

Nanopost	Type	1	2	3	4	5	6	7	8	9	10
Parameters	**θ**	40	-	10	40	-	-	40	20	70	40
	**b_1**	60	70	80	80	-	-	30	50	40	30
	**b_2**	70	80	80	100	-	-	60	-	50	40
	**r_1**	30	40	40	60	120	130	130	130	140	140
	**r_2**	-	-	-	-	60	70	-	-	-	-

S3: Measurement setup for characterizing the performances of broadband achromatic metalens and metalens array

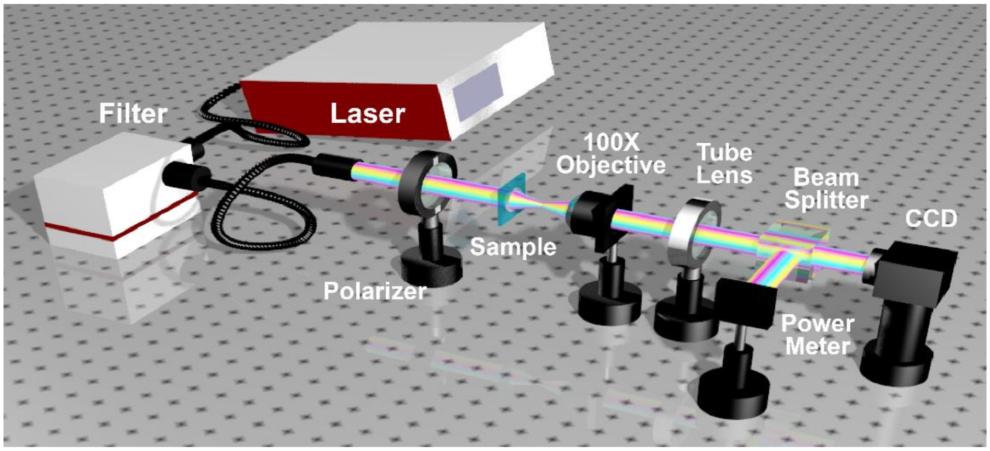

Figure S3. Measurement setup for characterizing the performances of broadband achromatic metalens and metalens array. The single achromatic metalens and metalens array are measured on the optical platform using a supercontinuum laser (Fianium WL-SC480-20-PP) with a filter (Fianium LLTF-VIS2-HP-FDS-SM). The laser beam with a linear polarization state is generated using a linear polarizer (OQGLC-15). An objective (100× magnification, NA = 0.95) is used to collect the transmitting light from the achromatic metalenses. The beam splitter and power meter are inserted to the light path for characterizing the focusing efficiency. Note that the adjustable iris (not shown in the illustration) between the tube lens and the beam splitter is used to limit the clear aperture.

S4: Calculation of the object film

As we all know, in the conventional integral imaging pickup process, the micro-lens array is used to capture the 3D light field information as the way of elemental image array (object film). Each micro-lens generates the corresponding elemental image (EI). We define the pixels in the same position from all EIs as the "homonymy points", and a set of "homonymy points" can be combined into the corresponding projected image, as shown in Fig. S4a. Thus inversely, in the actual pickup process, we can also directly capture the projected images and then use these projected images to generate the elemental image array (EIA).[1]

In the actual pickup method, we use the multiple orthographic frustum computer-generated integral imaging method[1-3] instead of the conventional optical integral imaging pickup method. As shown in Fig. S4b, we utilize the virtual camera array to capture the projected image array. After rendering through each virtual camera, the certain pixels from the obtained projected images are interleaved to be mapped on the certain positions of the elemental image array by using the selective pixel sampling algorithm[2,3]. The calculated equation can be expressed as,

$$E\left(x',y'\right)=I_{M-1-\mathrm{mod}\left(x',M\right),N-1-\mathrm{mod}\left(y',N\right)}\left(x'\frac{S_{\mathrm{V}}}{H_{\mathrm{EIA}}},\ y'\frac{R_{\mathrm{V}}}{V_{\mathrm{EIA}}}\right), \qquad \text{(S4.1)}$$

where E (x', y') is the pixel in the EIA's x'–y' plane, which belongs to a certain EI. And this pixel comes from the certain captured viewpoint image $I_{m,n}$ (x, y), where (m, n) represents the index of the virtual camera and the (x, y) represents the index of pixel in this corresponding viewpoint image. $H_{EIA} \times V_{\mathrm{EIA}}$ is the resolution of EIA. $S_V \times R_V$ is the resolution of the captured viewpoint image. $M \times N$ is the number of camera.

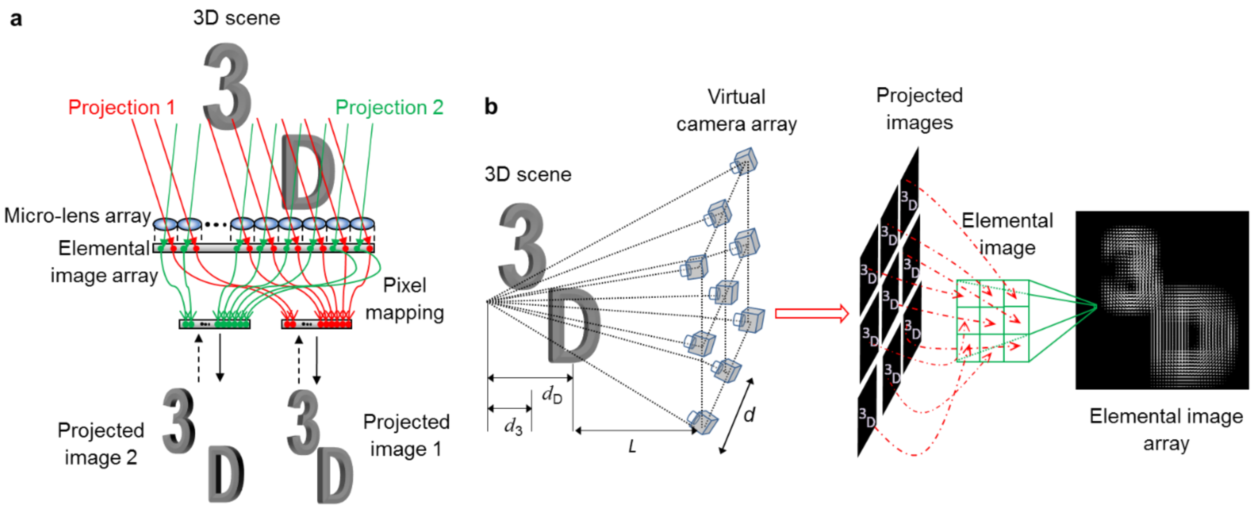

Figure S4. The calculation principle and the generation method of the object film. The object film is the elemental image array calculated by a computer based on the principle of the integral imaging display by micro-lens array. The number of the virtual camera array is $M \times N$. The distance between the camera array and the 3D scene (such as the model "**D**") is L and the distance of the adjacent cameras is d.

S5: Optical reconstruction processes of object films

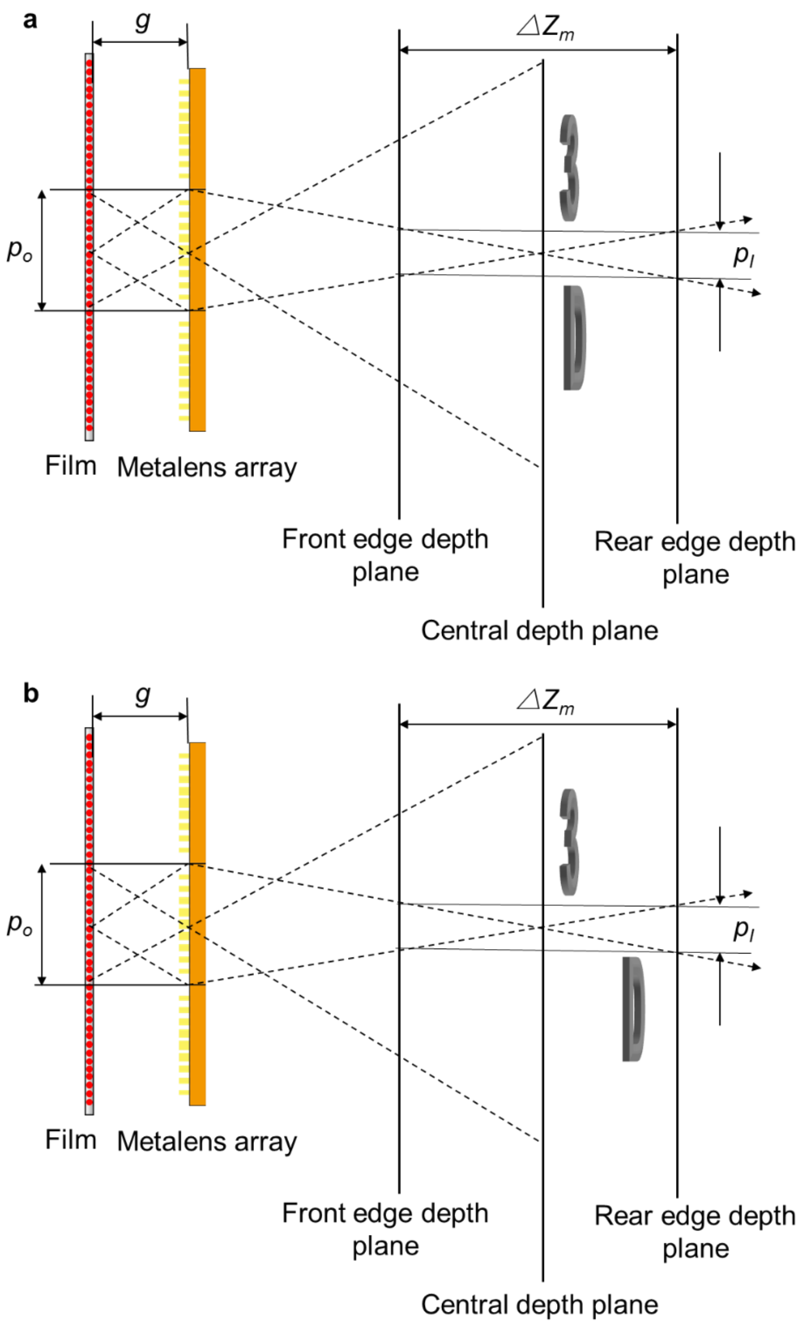

Figure S5. Optical reconstruction processes of object films. In the optical reconstruction process of integral imaging, the light from the film passes though the metalens array and the three-dimensional image is then reconstructed in the region around the central depth plane with an image depth of ΔZ_m. g is the distance between the metalens array and the film, p_0 is the size of micro-lens element, and p_I is the pixel size of the three-dimensional image. **a** The case of $d_D = d_3$. **b** The case of $d_D > d_3$.

In experiments, we move the microscopy imaging system along the optical axes to make the system focus from the plane of metalens to the rear edge depth plane. In the first case of $d_D = d_3$, number “3” and letter “D” are in the same depth plane (Fig. S5a), hence they will always become clear together. The results are shown in Fig 6b and Video m1. However, in the second case of $d_D > d_3$ (Video m2), since the number “3” is closer to the central depth plane than is the letter “D” (Fig. S5b), we can easily note that the number “3” first becomes clear while the letter “D” continues to blur (Fig. 6c), and then the letter “D” becomes clear while the number “3” will become blurred (Fig. 6d) compared with that in Fig. 6c. These imaging results greatly verifies the three-dimensional image depth effect using our metalens array in integral imaging.

S6: Optical reconstruction of the object film “D” using the 15×15 metalens array

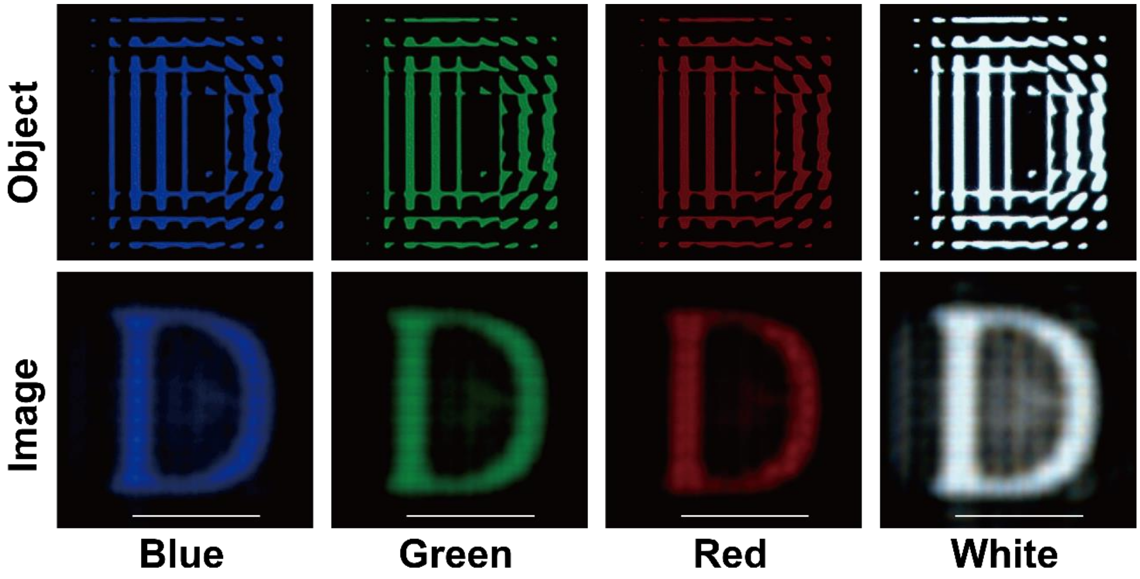

Figure S6. Imaging demonstration of the 15×15 broadband achromatic metalens array. The objects (up) illuminated by the blue, green, red and white light from the lamp and their corresponding images (bottom) through the metalens array. The blue, green and red light sources are generated by inserting the narrow-band (10 nm) filters of 488 nm, 532 nm and 633 nm respectively. Good-quality images, especially in the white light case, can be observed by adjusting the film and the metalens array to match each other. The white image explicitly shows the broadband achromatic property of metalens array in the entire visible region. Scale bar, 100 μm.

Reference

1 Li, S.-L., Wang, Q.-H., Xiong, Z.-L., Deng, H. & Ji, C.-C. Multiple Orthographic Frustum Combing for Real-Time Computer-Generated Integral Imaging System. *J. Display Technol.* **10**, 704-709 (2014).

2 Xing, Y., Wang, Q., Luo, L., Ren, H. & Deng, H. High-Performance Dual-View 3-D Display System Based on Integral Imaging. *IEEE Photonics Journal* **11**, 1-12 (2019).

3 Ren, H., Wang, Q.-H., Xing, Y., Zhao, M., Luo, L. *et al.* Super-multiview integral imaging scheme based on sparse camera array and CNN super-resolution. *Appl. Opt.* **58**, A190-A196 (2019).
